# A Multi-Site Validation in India of the Line Probe Assay for the Rapid Diagnosis of Multi-Drug Resistant Tuberculosis Directly from Sputum Specimens

**DOI:** 10.1371/journal.pone.0088626

**Published:** 2014-02-19

**Authors:** Neeraj Raizada, K. S. Sachdeva, D. S. Chauhan, Bharti Malhotra, Kishore Reddy, P. V. Dave, Yamuna Mundade, Pranav Patel, Ranjani Ramachandran, Ram Das, Rajesh Solanki, Douglas Fraser Wares, Suvanand Sahu, Rick O'Brien, C. N. Paramasivan, Puneet K. Dewan

**Affiliations:** 1 Foundation for Innovative New Diagnostics, New Delhi, India; 2 Central TB Division, Directorate General of Health Services, Ministry of Health and Family Welfare, Government of India, New Delhi, India; 3 National JALMA Institute for Leprosy and Other Mycobacterial Diseases, Agra, India; 4 Sawai Man Singh Medical College, Jaipur, India; 5 State TB Cell, Directorate of Health Services, Gujarat, India; 6 UNITAID, Geneva, Switzerland; 7 World Health Organization, New Delhi, India; 8 Global TB Programme, World Health Organization, Geneva, Switzerland; 9 Stop TB Partnership, Geneva, Switzerland; 10 Foundation for Innovative New Diagnostics (FIND), Geneva, Switzerland; University of Calgary & ProvLab Alberta, Canada

## Abstract

Rifampicin (R) and isoniazid (H) are key first-line anti-tuberculosis drugs. Failure to detect resistance to these two drugs early results in treatment failure and poor clinical outcomes. The study purpose was to validate the use of the GenoType MTBDR*plus* line probe assay (LPA) to detect resistance to R and H in *Mycobacterium tuberculosis* strains directly from smear-positive sputum samples in India.

**Method:**

Smear positive sputum specimens from 320 patients were subjected to LPA and results compared against those from conventional Lowenstein Jensen (LJ) culture and drug susceptibility testing (C&DST). All specimens with discordant R DST results were subjected to either sequencing of the rpoB gene and/or repeat DST on liquid culture (MGIT 960) at a National Reference Laboratory.

**Results:**

Significantly higher proportion of interpretable results were observed with LPA compared to LJ C&DST (94% vs. 80%, *p*-value <0.01). A total of 248 patients had both LJ and LPA DST results available; 232 (93.5%) had concordant R DST results. Among the 16 discordant R DST results, 13 (81%) were resolved in agreement with LPA results. Final LPA performance characteristics were sensitivity 96% (CI: 90%–98%), specificity 99% (CI: 95%–99%), positive predictive value 99% (CI: 95%–99%), and negative predictive value 95% (CI: 89%–98%). The median turnaround testing time, including specimen transportation time, on LPA was 11 days as compared with 89 days for LJ C&DST.

**Conclusions:**

LPA proved highly accurate in the rapid detection of R resistance. The reduction in time to diagnosis may potentially enable earlier commencement of the appropriate drug therapy, leading to some reduction of transmission of drug-resistant strains.

## Introduction

The global threat of multidrug-resistant tuberculosis (MDR-TB; strains of *Mycobacterium tuberculosis* resistant to at least rifampicin and isoniazid) to TB control, underscores the importance of prompt and rapid identification of such resistant *Mycobacterium tuberculosis* (*M.tb*) strains. Isoniazid (H) and rifampicin (R) are the key first-line anti-tuberculosis drugs, and resistance to these drugs i.e. MDR-TB, is likely to result in treatment failure and poor clinical outcomes [1], [2]

India has the largest number of estimated MDR-TB cases amongst notified TB patients of any country [3]. Up to the time of the study, the Government of India's Revised National TB Control Programme (RNTCP) had relied on conventional Lowenstein Jensen (LJ) culture and drug susceptibility testing (C&DST) for the diagnosis of drug resistant TB cases. By December 2009, there were however only 14 such laboratories across the country validated and certified by the RNTCP for conducting LJ C&DST [4].The mean time to detect drug resistance on egg based LJ media is around 3–4 months [5]. Even using the more modern broth-based liquid culture systems, C&DST results from sputum specimens still takes several weeks [6]. However, newly developed molecular based methods have advantages over conventional phenotypic methods in terms of both accuracy and turnaround time. The GenoType MTBDR*plus* assay is a commercially available line probe assay (LPA) from Hain Lifescience, Nehren, Germany, and is designed to simultaneously detect the most important gene mutations conferring R (*rpoB* genes) and H (*inhA, katG*) resistance in *M. tb* isolates within 8 hours [7].

A 2008 meta-analysis found that the GenoType MTBDR*plus* assay and another similar commercial test had a pooled sensitivity of 98% for detecting R resistance and 89% for detecting H resistance and specificity of 99% for R and H [8]. Testing can be performed on culture isolates or acid fast bacilli (AFB) positive sputum specimens, and can provide results within 8 hours, making this a promising tool to accelerate the diagnosis of MDR-TB cases, and hence improve management of MDR-TB cases.

Although the GenoType MTBDR*plus* assay has been studied in several laboratories, there is a wide variation in circulating *M.tb* strains across the globe [9], [10], and false negative results can occur due to the presence of unique genetic mutations in the different settings [8], [11]–[16]. Hence validation in different settings is needed to ensure acceptable performance. With its large number of MDR-TB cases, validation in India was deemed necessary ahead of wide-scale introduction of LPA for the programmatic management of drug resistant TB (PMDT) in the country.

To address this issue, a cross sectional study to evaluate the assay directly on sputum specimens was conducted under programmatic conditions in India by the Foundation for Innovative Diagnostics (FIND) in 2008–09. Three laboratories located in different regions of India and certified by the RNTCP for performing LJC & DST, were selected as the sites for the study. The primary objective of the study was to evaluate the accuracy of rifampicin and isoniazid susceptibility results by LPA performed directly on AFB smear-positive sputum specimens, compared against LJ C&DST. The secondary objective was to evaluate the operational performance characteristics of LPA versus LJ C& DST, specifically the time to reporting of test results and the proportion of invalid tests results.

## Materials and Methods

### Setting

The study was conducted at the public sector RNTCP state level Intermediate Reference Laboratories (IRL) in Hyderabad (Andhra Pradesh State), Ahmadabad (Gujarat State), and the Mycobacteriology laboratory at SMS Medical College, Jaipur (Rajasthan State). Smear-positive sputum specimens of TB patients from surrounding districts, who were failing on first-line anti-TB treatment, were routinely transported to these IRLs for LJ C&DST. The Ahmedabad and Hyderabad laboratories had been validated by a National Reference Laboratory (NRL) for conducting LJ C&DST. The Jaipur laboratory was undergoing validation at the time of the study. Hence the LJ C&DST for all specimens from this site was conducted at the National JALMA Institute for Leprosy and Other Mycobacterial Diseases Agra Uttar Pradesh State (NRL).

### Enrolment

Between November 2008 and January 2009, all patients submitting sputum specimens for LJ C&DST at the 3 study sites, were enrolled consecutively. As per RNTCP guidelines at the time of the study, MDR-TB suspects were defined as those TB patients who remained sputum smear positive after 4 months of treatment with an RNTCP Category 2 re-treatment regimen and who were not on treatment with any second-line anti-TB drugs (including any fluoroquinolone, ethionamide, cycloserine, PAS, or any second line injectable agent) at the time of specimen collection.

A cumulative sample size pre-specified for the study as per project protocol was approximately 250 specimens with both LPA and LJ C&DST results, across all sites. This was based on an estimated 30% prevalence of R resistance in tested specimens. The minimum acceptable performance parameter for LPA was pre-specified as detection of 95% of R resistance cases based on 5% precision.

Patients were excluded from enrolment into the study if specimens contained any preservative such as cetyl pyridinum chloride or if the sputum samples were from patients who had previously been confirmed as MDR-TB by any one of the standard laboratory procedures.

### Ethical considerations

The study was conducted in India, based on a memorandum of understanding between FIND and Government of India for the introduction of rapid new TB diagnostics in RNTCP of India. Accordingly, patients were managed as per organisational policy which was based on the results of LJ C&DST, as per routine. As LPA had not been validated for patient care in India and was not used for patient care prior to this study under RNTCP, the LPA results were not made available and hence not considered for decision making on patient care. As per the project protocol, as testing was conducted on remnant anonymised specimens, which otherwise be discarded and did not influence in any way patient management, informed consent was not considered necessary. The study protocol, after detailed review, was initially approved by the National laboratory committee constituted under RNTCP. The study protocol was further reviewed and approved independently by the ethical committees at each of the three study sites (namely the Ethics committee of the SMS Medical College and attached Medical College, and the Institutional Review Boards of the RNTCP State TB & Demonstration centres of Ahmedabad and Hyderabad).

### Laboratory procedures

#### Specimen collection and transportation

Two sputum specimens (one morning and one spot) were collected from all MDR-TB suspects in a pre-sterilised 50 ml centrifuge tube for LJ C& DST, in line with the programmatic guidelines. At the time of the specimen collection, a standard “request for C&DST form” was filled out by the respective laboratory staff. Specimens were transported within 7 days employing a cold chain to the reference laboratory without any preservative in the 50 ml centrifuge tube.

#### Specimen processing and LJ Culture and DST

Fresh sputum specimens were processed by N-acetyl-L-cystein-sodium hydroxide (Nalc-NaOH) method (with final NaOH concentration of 1%) as recommended by US Centers for Disease Control and Prevention CDC [17]. The concentrated sediment was re-suspended in 1–2 ml of phosphate buffer, subjected to Ziehl-Neelsen staining and inoculated on LJ media. After the growth on LJ slopes was obtained, isolates were subjected to *M.tb* complex identification by testing growth on para-nitro-benzoic acid medium, niacin test and nitrate reductase test [18]. All *M.tb* complex isolates were tested by 1% proportion method for drug susceptibility with critical drug concentration of isoniazid (0.2 µg/ml) and rifampicin (40 µg/ml) [19].

#### Line probe assay: DNA extraction

From each patient the specimen with the highest smear grading based on RNTCP guidelines [20] was tested by LPA. An aliquot of the processed sputum deposits was coded and assigned a unique study ID number for the LPA test. DNA extraction and amplification was performed as recommended by the manufacturer. Briefly, 0.5 ml of processed sputum deposit was centrifuged at 10,000 g for 15 minutes, re-suspended in 100 µl of molecular grade water, sealed and heated for 20 minutes at 95°C in a water bath followed by ultrasonication for 15 min at room temperature. This suspension was centrifuged at 13,000 g for 5 minutes, and the supernatant (DNA Extract) transferred by pipette to a fresh tube without disturbing the pellet. A 5 µL aliquot of this extracted DNA was used for amplification procedures. The Genotype MTBDR*plus* assay version.1 was performed as recommended by the manufacturer [7].

#### PCR amplification

Amplification was performed by combining 35 µL of primer nucleotide mix supplied by the manufacturer (PNM) with 5 µL of 10× PCR buffer (containing 15 mM MgCl2), 2 µL MgCl2 (25 mM MgCl2), 3 µL molecular grade H2O, 0.2 µL (1 unit) Hot-Star *Taq* polymerase (QIAGEN, Hilden, Germany), and 5 µL of the DNA for a total final volume of 50.2 µL. The amplification profile for direct patient material as described by the manufacturer was used for all sputum specimens. First, the template DNA was denatured for 15 minutes at 95°C, followed by 10 cycles consisting of 30 seconds at 95°C and 2 minutes at 58°C, with an additional 30 cycles consisting of 25 seconds at 95°C, 40 seconds at 53°C and 40 seconds at 70°C. The final cycle consisted of an 8 minute run at 70°C.

#### Hybridization and Detection

Hybridization was performed using the hybridization Kits, including reagents and 12 well plastic tray and instrument (Twincubator) as provided by the manufacturer [7]. Briefly, 20 µl of denaturation solution (DEN,blue) were mixed thoroughly in a plastic 12-well tray, with 20 µl of amplified sample (PCR product) and incubated at room temperature for 5 minutes. After denaturation, the biotin-labelled amplicons were hybridized (using HYB, green solution) to the single stranded membrane-bound probes. After a stringent washing (using STR-red solution), a streptavidinalkaline phosphatase conjugate (1∶100 dilution of Con-C with CON-D) was added to the strips and an alkaline phosphatase-mediated staining reaction (1∶100 dilution of SUB-C with SUB-D) was observed in the bands where the amplicon and the probe had hybridized [7].

#### Interpretation of results

The MTBDR*plus* assay strip contains 27 reaction zones; 21 of them are probes for mutations and 6 are control probes for verification of the test procedures. The six control probes include a conjugate control, and amplification control, an *M.tuberculosis* complex-specific control (TUB), an *rpoB* amplification control, a *katG* amplification control, and an *inhA* amplification control. For the detection of R resistance, the probes cover the *rpoB* gene, while the H resistance specific probes cover positions in *katG* and *inhA*. The absence of at least one of the wild-type bands or the presence of bands indicating a mutation in each drug resistance-related gene implies that the sample tested is resistant to the respective antibiotic. When all the wild-type probes of a gene stain positive and there is no detectable mutation within the region examined, the sample tested is susceptible to the respective antibiotic. In order to give a valid result, all six expected control bands should appear correctly. Otherwise, the result is considered invalid. [7].

#### Repeat testing

Sputum specimens resulting in inconsistent development of bands on the MTBDR*plus* strip and/or if no *M.tb* control band appeared, underwent repeat PCR and hybridization from the extracted DNA. Isolates with discordant results of the LPA and the LJ C&DST, were sent to a national reference laboratory (JALMA Institute, Agra) for sequencing of *rpoB* and repeat C& DST testing using liquid (MGIT 960) culture systems. Sequencing of discordant results sample was performed using BigDye Terminator v3.1 Cycle Sequencing Kit and analyzed on an ABI 3130xl genetic analyzer (Applied Biosystems, USA). Sequencing results were compared with the DNA sequences of wild type reference strain *M. tuberculosis* H37Rv using MegAlign program (DNASTAR, Madison, WI, USA).

#### Data Analysis

The sensitivity, specificity, predictive value positive (PPV), predictive value negative (NPV), and overall accuracy of LPA results were compared to the conventional LJ DST results for R and H, and the ability of R resistance alone to predict MDR. For calculation, the reference DST result for R was the LJ DST result for specimens with initially concordant LPA and LJ DST results, and the results of the repeat testing procedure described above for those specimens with initially discordant LJ and LPA DST results. An analysis of banding patterns associated with R and H resistance in MDR-TB and non MDR-TB strains was performed. Statistical tests to assess the test performance used under the study were odds ratio, sensitivity, specificity, positive predictive value, and negative predictive value. These were calculated using free online statistical calculators available at http://www.medcalc.org/calc/.

## Results

Between November 2008 and January 2009, sputum specimens from 320 sputum smear positive MDR-TB suspects were received from the pre-identified districts as per the study protocol, and entered into the study. Only 1 specimen of the 2 received per patient was tested on LPA, with the specimen showing the better growth on LJ media tested for susceptibility to R and H.

Of the 320 patients, 72% (230/320) were male, and the mean age was 37.1 years. Testing of all 640 samples by culture yielded at least one positive growth for *M.tb* complex in 256 (80%) patients. Samples from 51 (16%) patients had no growth and in 13 (4%) patients the cultures were contaminated. LPA gave interpretable results for 301 (94%) patients. LJ DST results were available for all 256 patients with positive culture results (table 1).

**Table 1:** Proportion of invalid LPA results in comparison to culture results.

	LJ Positive	LJ No Growth	LJ Contaminated	Total
**Valid LPA**	248	41	12	301 (94%)
**Invalid LPA**	8	10	1	19 (5.9%)
**Total**	256 (80%)	51 (16%)	13 (4%)	320

Overall, a significantly higher proportion of patients had interpretable results from LPA as compared to LJ culture and DST (94% vs. 80%, *p*-value <0.01). Amongst the 51 culture negative patients, 41 (80%) had a valid LPA result, and 12 (92.3%) of the 13 patients with contaminated cultures had an interpretable LPA result (Table 1). There was a significantly higher likelihood of obtaining an interpretable MTBDR*plus* result from a specimen with a positive smear grade compared to those specimens with a scanty smear grading (*p*-value 0.012) (Table 2).

**Table 2:** Association between smear positivity grades and LPA test result.

	Smear Grade							
	Scanty		1+		2+		3+	
	No	%	No	%	No	%	No	%
**Valid LPA**	6	17	5	5	6	8	2	2
**Invalid LPA**	30	83	105	95	70	92	96	98
**Total**	36		110		76		98	
**p-value: 0.012**								

### DST results

The median time from the specimen collection to the LJ C&DST result being available was 87 days (range of 42 to 208 days). In comparison, the median time to obtain a LPA result was just 11 days (range of 1 to 76 days). This time included specimen shipment time which varied from 1 to 7 days.

**Figure 1:**
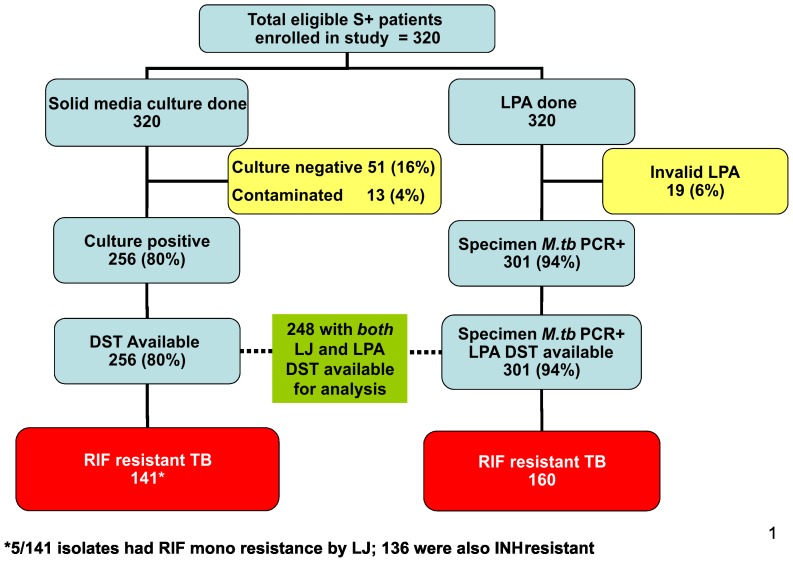
Suspect enrolment and result summary.

In this patient population of consecutively-enrolled smear-positive TB patients suspected of having MDR-TB, the prevalence of rifampicin resistance was high. Among the 256 patients with available LJ DST results, 136 (53%) were resistant to both R and H i.e. MDR-TB, 5 (2%) were resistant to R and susceptible to H, 54 (21%) were resistant to H and susceptible to R and 61 (24%) were susceptible to both R and H. Among the 301 patients with interpretable LPA results, 127 (42%) were MDR-TB, 33 (11%) had R mono-resistance, 30 (10%) were resistant to H and susceptible to R, and 111 (37%) were susceptible to both R and H (Figure 1). Overall, a total of 141 patients were detected with R-resistance by LJ media, whereas 160 were detected by LPA i.e. an additional 19 (6%) R-resistant cases were identified by LPA.

### Drug resistance associated mutations

The most common mutation detected by LPA in the rpoB gene was S531L (47%), diagnosed by the presence of MUT3 band. There was no significant difference in the prevalence of this mutation in MDR-TB specimen and R mono-resistance specimen (47% vs 46%). Four (2.7%) cases had multiple mutations - 2 had D516V and S531L mutations, one had H526Y and H526D mutations, and one had H526D and S531L mutations. Of the overall 160 patients in whom R-resistance was detected, 60 were on the basis of missing Wild type probes and did not have any positive mutant probe (table 3).

**Table 3:** Gene mutation patterns in resistant M.tb strains using Genotype MTBDRplus LPA.

Gene	Band	Gene Region or Mutation	MDR (n = 127)	H monoresistance (n = 30)	R monoresistance (n = 33)
***rpoB***	WT1	506–509	126 (99)	30 (100)	33 (1 00)
	WT2	510–513	121 (95)	30 (100)	31 (94)
	WT3	513–517	104 (82)	30 (100)	28 (85)
	WT4	516–519	104 (82)	30 (100)	29 (88)
	WT5	518–522	125 (98)	30(100)	33 (100)
	WT6	521–525	122 (96)	30 (100)	32 (97)
	WT7	526–529	101 (80)	30 (100)	27 (82)
	WT8	530–533	50 (39)	30 (100)	12 (36)
	MUT1	D516V	11 (9)	0 (0)	4 (12)
	MUT2A	H526Y	8 (6)	0 (0)	2 (6)
	MUT2B	H526D	4 (3)	0 (0)	0 (0)
	MUT3	S531L	60 (47)	0 (0)	15 (46)
***katG***	WT	315	19 (15)	8 (27)	33 (100)
	MUT1	S315T1	97 (76)	15 (50)	0 (0)
	MUT2	S315T2	0 (0)	0 (0)	0 (0)
***inhA***	WT1	0.9375	110 (87)	28 (93)	33 (100)
	WT2	−8	110 (87)	26 (87)	33 (100)
	MUT1	C15T	9 (7)	3 (10)	0 (0)
	MUT2	A16G	0 (0)	0 (0)	0 (0)
	MUT3A	T8C	2 (2)	0 (0)	0 (0)
	MUT3B	T8A	0 (0)	1 (3)	0 (0)

Definition of abbreviations: H = isoniazid; MDR = multidrug-resistant; R = rifampicin.

Values are numbers, with percentages in parentheses.

### Concordance between LJ DST and LPA DST

A total of 248 patients had both LJ and LPA DST results available. Initial analysis showed agreement of results in 232 (94%) patients, including 127 with R-resistance and 105 with R-susceptibility on both LPA and LJ media (initial concordance 94%; Sensitivity: 93% (CI: 88%–96%); Specificity: 94% (CI: 88%–97%) Positive Predictive value: 95% (CI: 90%–97%) Negative Predictive value: 92% (CI: 86%–96%) (Table 4). There were 16 (6%) specimens with discordant rifampicin DST results between LJ and LPA. These included 9 results that were R-resistant on LJ and susceptible on LPA, and 7 that were R-susceptible on LJ and resistant on LPA (table 4). Each of these 16 specimen were subjected to sequencing of *rpoB* gene and repeat Culture and DST at the national reference laboratory as per the repeat testing procedure. Of these, 11 specimens could be sequenced, and culture and DST results of 15 results were available at the end of the study. For specimen that could not be either sequenced or have a valid result on culture and DST at national reference laboratory, LJ C&DST being the pre-existing procedure, was presumed to be correct. The repeat testing procedure agreed with the LPA result in 12 instances (including one mixed infection) and the LJ media result in 3 instances. One test could not be performed due to heavy contamination found in the MGIT DST (table 5).

**Table 4:** Correlation of R and H resistance on LJ DST with LPA.

Rifampicin Resistance				Isoniazid Resistance			
	LJ DST Rifampicin Resistant	LJ DST Rifampicin Sensitive	Total Results		LJ DST Isoniazid Resistant	LJ DST Isoniazid Sensitive	Total Results
LPA Rifampicin Resistant	127	7	134	LPA Isoniazid Resistant	133	2	135
LPA Rifampicin Sensitive	9	105	114	LPA Isoniazid Sensitive	52	61	113
Total Results	136	112	248	Total Results	185	63	248
**Concordance: 94%**				**Concordance: 78%**			
**Sensitivity: 93% (CI: 88%–96%)**				**Sensitivity: 72% (CI: 65%–78%)**			
**Specificity: 94% (CI: 88%–97%)**				**Specificity: 97% (CI: 89%–99%)**			
**Positive Predictive value: 95% (CI: 90%–97%)**				**Positive Predictive value: 99% (CI: 95%–99%)**			
**Negative Predictive value: 92% (CI: 86%–96%)**				**Negative Predictive value: 54% (CI: 45%–63%)**			

**Table 5:** Results of discordance resolution at national reference laboratory.

Specimen Number	Rifampicin LJ C& DST	LPA result for Rifampicin Resistance	Sequencing result at National Reference Laboratory	Culture and DST at National Reference Laboratory	Final Interpretation
**A**	Resistant	Sensitive	-	Contaminated	Resistant
**B**	Resistant	Sensitive	-	Sensitive	Sensitive
**C**	Resistant	Sensitive	-	Sensitive	Sensitive
**D**	Resistant	Sensitive	Sensitive	Sensitive	Sensitive
**E**	Resistant	Sensitive	Sensitive	Sensitive	Sensitive
**F**	Resistant	Sensitive	-	Sensitive	Sensitive
**G**	Resistant	Sensitive	-	Sensitive	Sensitive
**H**	Resistant	Sensitive	Resistant	Resistant	Resistant
**I**	Resistant	Sensitive	Sensitive	Resistant	Resistant
**J**	Susceptible	Resistant	Resistant	Sensitive	Resistant
**K**	Susceptible	Resistant	Resistant	Resistant	Resistant
**L**	Susceptible	Resistant (Mixed Infection)	Sensitive	Resistant	Mixed Infection
**M**	Susceptible	Resistant	Resistant	Resistant	Resistant
**N**	Susceptible	Resistant	Resistant	Resistant	Resistant
**O**	Susceptible	Resistant	Resistant	Resistant	Resistant
**P**	Susceptible	Resistant	Resistant	Resistant	Resistant

Based on the final DST results, i.e. the LJ DST adjusted for repeat testing conducted at national reference laboratory, LPA detected R-resistance with concordance 97%, sensitivity, 96% (CI: 90%–98%), specificity, 99% (CI: 95%–99%), positive predictive value, 99% (CI: 95%–99%) and negative predictive value, 95% (CI: 89%–98%). (Table 6)

**Table 6:** Final reconciled results of LPA-LJ Rifampicin resistance correlation in view of sequencing and Liquid Culture and DST at National reference laboratory.

	LJ C&DST reconciled with liquid culture & DST/sequencing at national reference laboratory		
	Resistant	Sensitive	Total
**LPA Rifampicin Resistant**	**133**	**1**	**134**
**LPA Rifampicin Sensitive**	**6**	**108**	**114**
**Total**	**139**	**109**	**248**
**Concordance: 97%**			
**Sensitivity: 96% (CI: 90%–98%)**			
**Specificity: 99% (CI: 95%–99%)**			
**Positive Predictive value: 99% (CI: 95%–99%)**			
**Negative Predictive value: 95% (CI: 89%–98%)**			

In relation to H, the analysis showed agreement of results in 194 (78%) patients, including 133 with H-resistance and 61 with H-susceptibility on both LPA and LJ media (Sensitivity: 72% (CI: 65%–78%); Specificity: 97% (CI: 89%–99%); Positive Predictive value: 99% (CI: 95%–99%) Negative Predictive value: 54% (CI: 45%–63%). There were 54 (22%) specimens with discordant H DST results between LJ C&DST and LPA (table 4).

## Discussion

The current study demonstrated that overall sensitivity and specificity of LPA assay for detection of R resistance was high at 96% and 99% respectively. This was similar to previously reported results in studies from South Africa, Germany, and Italy [21]–[28]. However, sensitivity and specificity of LPA assay for detection of H, resistance were 72% and 97% respectively. The sensitivity to detect H resistance is somewhat lower than previously reported results [8], [28].

The study findings suggest that LPA is suitable for routine use in settings where a standardised second line anti-TB drug regimen is provided to MDR-TB cases and where R-resistance is also treated with the same standardised MDR-TB regimen. Patients tested were more likely to have valid results available with LPA to guide clinical action, compared to LJ; this reflected reduced recovery of viable *M. tuberculosis* and culture contamination with LJ. This translated to increased detection of drug-resistant TB. With the observed 99% specificity of the LPA assay in detecting R-resistance in M.tb isolates, most of the patients would be appropriately treated with the standardised MDR-TB treatment regimen if this test were to be used for the routine and rapid diagnosis of R-resistance and MDR-TB. This is further reaffirmed by the results of LJ DST in the study, which showed that R mono-resistance was relatively rare.

As reported widely elsewhere, phenotypic rifampicin resistance was strongly associated with mutation in the 81 base pair region of rpoB targeted in the LPA assay [24], [25]. In this study, the most commonly observed mutations were in the region of *rpoB* 530–533, mostly S531L mutation. This is similar to the findings of in a South African study [15].

In this patient population of consecutively-enrolled smear-positive TB patients suspected of having MDR-TB, the prevalence of rifampicin resistance was unsurprisingly high. While using LPA in settings with significantly lower levels of MDR-TB, routine implementation of quality assurance guidance issued by agencies such as Global Laboratory Initiative (GLI) would be of paramount importance to address any concerns of false negative results.

The routine use of LPA can substantially reduce the time to diagnosis of R and/or H-resistant TB, and can hence potentially enable earlier commencement of appropriate drug therapy and thereby facilitate prevention of further transmission of drug-resistant strains. This confers a major advantage to this test. Until the time of the study, India had no access to newer diagnostic tests in its public funded TB laboratories and relied largely on LJ based solid C&DST. Unacceptable delays in obtaining both culture and DST results by these conventional methods were commonplace, specifically in drug resistant cases. A number of patients may be “lost” due to default and/or death whilst awaiting the availability of the DST results. The study also highlights that the availability of rapid diagnostics at central laboratories needs to be supplemented with rapid specimen transportation mechanisms.

### Conclusion

The multi-centric study reported here evaluated the performance of the Genotype MTBDR*plus* for the detection of R and H resistance under routine conditions in 3 state level reference laboratories in India, and provided direct evidence on the accuracy and feasibility of LPA. The GenoType MTBDR*plus* LPA version 1 assay is a sensitive and specific tool for the detection of rifampicin resistance in AFB smear-positive sputum specimens. The relatively quick turnaround time and the potential avenue for rapid screening of a large number of specimens/patients make it suitable as a first-line molecular diagnostic test for rifampicin resistance in settings such as India. The results of this evaluation were presented to the National TB Laboratory Committee of India in July 2009, which endorsed the routine use of the GenoType MTBDR*plus* LPA in the national TB programme for the testing of MDR–TB suspects [29], [30].
